# Zika Virus Vaccines: Challenges and Perspectives

**DOI:** 10.3390/vaccines6030062

**Published:** 2018-09-13

**Authors:** Raquel das Neves Almeida, Trina Racine, Kelly G. Magalhães, Gary P. Kobinger

**Affiliations:** 1 Laboratory of Immunology and Inflammation, University of Brasilia, Brasilia 70910-900, Brazil; raquel.das-neves-almeida.1@ulaval.ca (R.d.N.A.); kellymagalhaes@gmail.com (K.G.M.); 2 Centre de Recherche en Infectiologie du CHU de Québec—Université Laval, Québec, QC G1V 4G2, Canada; trina.racine@crchudequebec.ulaval.ca; 3 Department of Medical Microbiology, University of Manitoba, Winnipeg, MB R3E 0J9, Canada; 4 Department of Immunology, University of Manitoba, Winnipeg, MB R3E 0T5, Canada; 5 Départment de Microbiologie-Infectiologie et D’immunologie, Université Laval, Québc, QC G1V 0A6, Canada; 6 Department of Pathology and Laboratory Medicine, University of Pennsylvania School of Medicine, Philadelphia, PA 19104-4238, USA

**Keywords:** zika virus, flaviviruses, vaccines, animal models, immunopathology, immune responses, challenge, clinical trials

## Abstract

Zika virus is an arbovirus that has rapidly spread within the Americas since 2014, presenting a variety of clinical manifestations and neurological complications resulting in congenital malformation, microcephaly, and possibly, in male infertility. These significant clinical manifestations have led investigators to develop several candidate vaccines specific to Zika virus. In this review we describe relevant targets for the development of vaccines specific for Zika virus, the development status of various vaccine candidates and their different platforms, as well as their clinical progression.

## 1. Introduction

*Flavivirus* is a genus of viruses in the family *Flaviviridae* that includes *Dengue virus* (DENV), *Yellow fever virus* (YFV), *West Nile virus* (WNV), and *Zika virus* (ZIKV), all of which are transmitted by arthropod vectors [1,2,3]. However, ZIKV presents with additional modes of transmission to that of mosquito bites via vertical, sexual, and transfusion transmissions [4,5,6,7].

ZIKV was originally discovered in a sentinel macaque in the Zika forest in Uganda in 1947, while the first reported case of human infection was identified in 1952 [8]. Thereafter, sporadic infections were reported until the Micronesia outbreak of 2007. Symptoms reported as part of this outbreak included rash, conjunctivitis, and arthralgia [9]. Six years later, another outbreak occurred in French Polynesia and the first cases of hospitalization due to Guillain–Barré syndrome (GBS) were reported [10]. From 2015 onward, several cases of ZIKV infection were detected in Brazil, which then spread to other South American countries, leading the World Health Organization (WHO) to declare ZIKV a public health emergency of international concern (PHEIC) on 1 February 2016, a status subsequently lifted on 18 November 2016 [11,12].

**Figure 1:**
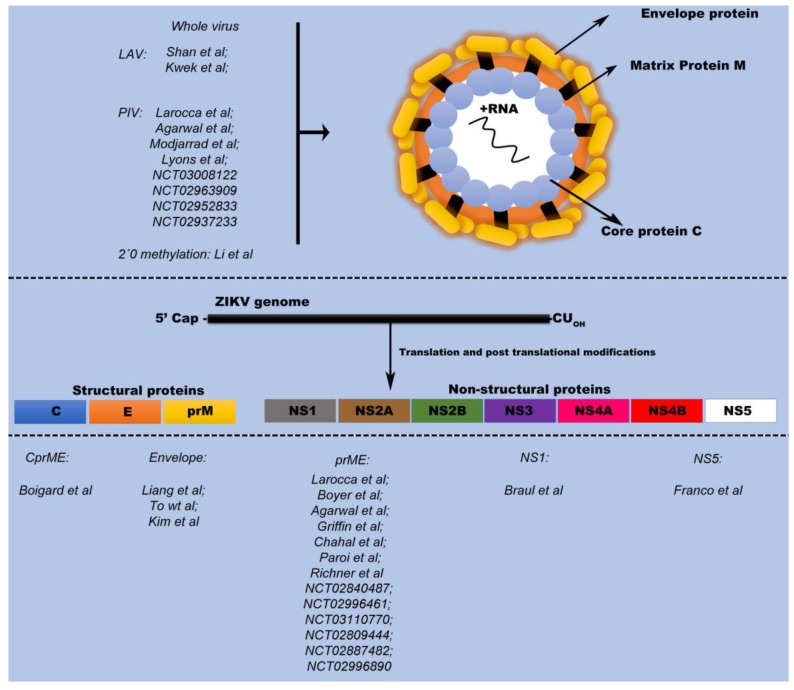
Various vaccines developed against Zika virus and their respective targets. LAV: Live Attenuated Virus; PIV: Purified Inactivated Virus.

The most recent outbreak of ZIKV was associated with congenital malformation and fetal/newborn microcephaly caused by ZIKV infection during pregnancy [13]. Furthermore, additional clinical manifestations were associated with ZIKV infection including neurological disorders and male infertility in animal models [14,15]. Due to the presence of these clinical complications, rapid scientific investigations began with the goal of better understanding viral pathogenesis, host response, and animal models of ZIKV infection. The global scientific effort to develop vaccines was also accelerated at this time with the goal of preventing or stopping ZIKV spread. However, to design an efficacious ZIKV vaccine it is necessary to select an efficient platform, evaluate the costs, choose the best specific immunogen, and test the vaccine’s immunogenicity and protective efficacy [16,17,18]. The ZIKV vaccines currently under development use different targets and platforms, and some of these vaccines are already undergoing clinical testing (Figure 1).

While ZIKV consists of a single serotype, there exist several lineages and as such, immunogen selection requires particular attention owing to the high structural similarity within the family *Flaviviridae*. This structural similarity between ZIKA virus and other *Flavivirus* could possibly lead to severe complications upon secondary *Flaviviridae* infection due to the mechanisms of neutralization and the potential for antibody-dependent enhancement (ADE) [19,20], although this has not been observed in nature.

Immunogen and vaccine platform selection of the ZIKV vaccine candidates currently undergoing clinical testing will be topics raised and discussed in this review.

## 2. ZIKV Vaccine Targets and Immunogens

The ZIKV genome is comprised of an ~11 kb single-stranded positive sense RNA genome encoding three structural proteins: capsid (C); precursor membrane (prM); and envelope (E), as well as seven nonstructural proteins NS1; NS2A; NS2B; NS3; NS4A; NS4B; and NS5. While the prME proteins are the main immunological targets for vaccine development [21], a few groups have developed vaccines that target some of the nonstructural proteins [22,23]. A major focus in vaccine development is identifying an optimal antigen for the induction of a robust immune response, often with the goal of eliciting a robust neutralizing antibody (NAb) response capable of neutralizing the virus and ultimately protecting against infection. Due to their ability to induce a robust immune response, the prME proteins are the viral antigens most frequently present in ZIKV vaccines. Three vaccine platforms, DNA-, mRNA-, and Adenovirus-based, are in the most advanced stages of development and all primarily use the prME antigens for the induction of the aforementioned neutralizing Ab response.

### 2.1. ZIKV Structural Proteins: Envelope Protein

The major structural component on the ZIKV surface is the E protein, which has been shown to induce high titers of neutralizing antibodies [24]. The E protein is composed of three structural domains, EDI, EDII, and EDIII, with EDI stabilizing the orientation of the protein and EDII promoting virus-mediated membrane fusion. EDII is located at the C-terminus, contains antigenic epitopes against which several NAbs have been identified and potentially is involved in binding to cellular receptors [24,25,26,27,28].

Liang et al. used *E. coli* and *Drosophila* as expression systems to produce recombinant ZIKV E protein. Following immunization of immunocompetent mice with recombinant ZIKV E protein, they observed that their protein immunization regimen generated a T-cell response and NAbs. They proposed that the vaccine could be used alone or in combination with other forms of vaccination [29]. To et al. using a similar recombinant protein strategy, obtained ZIKV E protein from *Drosophila* S2 cells and demonstrated potent T-cell responses against ZIKV in Swiss Webster (SW) BALB/c (Th2-dominant) and C57BL/6 (Th1-dominant) mouse models [30]. Kim et al., developed an adenovirus-based vaccine, Ad5.ZIKV-Efl, containing a portion of the ZIKV BeH815744 E protein fused to a T4 fibritin foldon trimerization domain (ZIKV-Efl). The Ad5.ZIKV-Efl vaccine generated a potent response in neonatal mice in vertical immunization trials [31]. Yang et al., developed a virus like particle (VLP) HBcAg-zDIII VLP, that is based on the hepatitis B core antigen (HBcAg) and expresses the ZIKV EDIII structural domain. Two immunizations of C57BL/6 mice was sufficient to elicit a potent cellular and humoral immune response that led to protection against multiple ZIKV strains. In addition to the apparent protective efficacy of the HBcAg-zDIII VLP vaccine, the HBcAg-zDIII-elicited antibodies did not enhance DENV infection in Fc gamma receptor-expressing cells [32].

### 2.2. ZIKV Structural Proteins: prME

In the *Flavivirus* virion, the E glycoprotein forms a complex with the prM glycoprotein and together they play an important role in virion maturation and in the virus life cycle [33,34,35]. Virion assembly and protein glycosylation occurs within the Endoplasmic Reticulum, while the *trans*-Golgi network (TGN) is responsible for virion maturation. The prME heterodimers found in the immature virion initial formed in the ER are transported to the TGN where, due to the acidic pH, the envelope protein undergoes structural changes allowing the cellular protease furin to cleave prM into pr and M, thus leading to the mature virion [36,37,38].

Several articles have demonstrated that these complexes induce high Ab titers, which has led to prME being the most predominant immunogen for the development of ZIKV vaccines [39,40,41]. ZIKV prME DNA- and RNA-based vaccines demonstrate consistent results in the induction of a protective immune response.

Emanuel et al. generated two distinctive vesicular stomatitis virus (VSV) based ZIKV vaccines where either the codon-optimized sequence of the full-length prME (ZIKVprME) or prM and soluble envelope (ZIKVprMsolE) were introduced into the VSV-EBOV vaccine vector backbone. Both vaccines were effective at delivering and co-expressing ZIKV and EBOV antigens and both vaccines conferred protection against lethal ZIKV challenge in the IFNAR^−/−^ mouse model 28 days after administration. In addition, the VSV-ZIKVprME vaccine was capable of conferring full protection when administered as little as three days prior to a lethal ZIKV challenge [42].

The MV-ZIKV101 vaccine developed by Themis Bioscience, constructed using a measles virus vector backbone expressing the prME proteins, is currently in clinical trials (NCT02996890). Indeed, all ZIKV vaccine candidates in clinical trials contain prME, either as a component of a whole virion or as an immunogen. Furthermore, prME based vaccines against other flaviviruses have shown great potential, resulting in approval and commercialization in some countries [43,44].

### 2.3. Nonstructural Protein: NS1

The nonstructural NS1 glycoprotein is an internal protein secreted during viral replication. NS1 is highly immunogenic and has been used in DNA-based vaccines for other flaviviruses, making it another potential target for ZIKV vaccine development [45,46]. The plasmids pcTPANS1 and pDEN-2-NS1 containing the NS1 sequence from DENV2 provided protection against lethal DENV2 challenge with specific cellular and humoral responses being shown in BALB/c C3H mice [47,48]. Brault et al., developed a ZIKV vaccine based on NS1 by inserting the ZIKV NS1 sequence from the Suriname 2015 Asian isolate into a Modified Vaccinia Ankara (MVA) vector resulting in the generation of MVA-ZIKV-NS1. A single intramuscular (IM) dose of MVA-ZIKV-NS1 administered to wild type mice (CD-1/ICR) generated both humoral and cellular immune responses providing total protection against a lethal ZIKV challenge [22]. Despite these encouraging results, immunization with the NS1 antigen requires additional evaluation to determine the vaccine’s safety since studies conducted on DENV NS1 have demonstrated the potential for anti-NS1 Abs to possess cross-reactivity with specific host proteins leading to platelet aggregation and cell death [49,50].

### 2.4. Nonstructural Protein: NS5

The nonstructural NS5 *Flavivirus* glycoprotein is consider essential for RNA genome replication because it possess an N-terminal methyltransferase necessary for RNA capping [51,52,53]. A single report discusses the design of a NS5 antigen potentially capable of inducing a protective cellular immune response against both ZIKV and DENV [21]. Here, using in silico predictive algorithms, the 19 epitopes selected are highly conserved between DENV and ZIKV and are predicted to be able to induce cross-protection among different populations around the world. However, in order to verify its potential as a cross-protective immunogen, in vivo studies need to be performed.

### 2.5. Others Strategic Targets

In vaccine development, the requirement for a safe, effective and straightforward immunization method is paramount. Boigard et al. proposed using VLPs to recreate the immunological determinants of ZIKV while remaining noninfectious. ZIKV VLPs were produced through the co-expression of the Capsid, pre-Membrane, and Envelope (CprME) and NS2B/NS3 proteins in mammalian cells. Mice immunized with these VLPs generated high Ab titers and exhibited greater survivability when compared to mice vaccinated with inactivated virus [54]. This is possibly due to variation in capsid structure owing to the inactivation process [54,55,56].

Another broad strategy proposed for all *Flaviviridae* members consists of generating a recombinant virus defective in 2′-O methylation, as proposed by Li and colleagues [57]. The 2′-O methylation process mimics the methylation of host mRNA, thus promoting the replication of the viral genetic material. Defective 2′-O methylation permits brief periods of replication, however, viral RNA recognition by host innate immunity prevents long term replication within cells. Li et al., generated a 2′-O methylation defective Japanese encephalitis virus (JEV) that generated both humoral and cellular responses in BALB/c mice, conferring protection against subsequent lethal challenges of JEV [57]. This promising approach may yet yield a ZIKV vaccine using 2′-O methylation defective ZIKV.

## 3. ZIKV Vaccines and Platforms in Human Clinical Trials

The most commonly used vaccination technology is based on the live attenuated virus (LAV) platform. The LAV platform takes advantage of viral strains, weakened by mutations, to induce an immune response without causing a full-blown infection. Producing LAVs is relatively cheap compared with other platforms that require adjuvant formulations and additional downstream processing to obtain a highly purified product. Moreover, LAVs generate highly specific and long-lasting immune responses. To develop a LAV platform against ZIKV, Sha et al., proposed an attenuating mutation that included the elimination of the final 10 nucleotides from the 3’ untranslated region (UTR) of the ZIKV genome (ZIKV-3’UTR-LAV). This strategy has shown efficacy in immunocompromised (AG129 mice) and immunocompetent (CD-1 mice) mouse models, as well as in non-human primate (NHP) models of infection [58]. Vaccination of rhesus macaques generated low levels of neutralizing antibody titers that were enhanced after ZIKV challenge, suggesting incomplete protection against ZIKV. Nevertheless, the ZIKV-3’UTR-LAV demonstrated that a single dose of vaccine was capable of preventing pathological damage in testes and vertical transmission [59]. Kweket et al. also proposed a new approach to attenuate ZIKV by serial in vitro passage, thereby selecting an attenuated ZIKV variant [60].

Development of LAV vaccines requires careful attention as reversion events may be possible, making it not as safe as other platforms. For this reason, the WHO cautions the use of LAVs in high-risk groups, such as fertile and pregnant women and immunocompromised individuals [61]. Due to the potential for reversion, additional validation and further in vivo evaluation is required to develop a suitable LAV candidate.

### 3.1. Inactivated Whole Virus Vaccine

Purified inactivated virus (PIV) vaccines consist of inactivated pathogens that are killed by heat or chemical agents, such as formalin, to reduce their virulence. Thereafter, the virus particles are purified and ready to be used for immunization. PIV vaccines contain whole virus that cannot replicate owing to their inactivation. Through the inactivation process, protein antigens remain intact, enabling recognition by the host immune system. However, for long-term protection, the use of an adjuvant might be required to boost the chronicity and immunogenicity of the antigen and strengthen the subsequent immune response [62,63]. However, according to the WHO, adjuvant administration during pregnancy needs to be considered on a case-by-case basis due to the safety profile of various potential adjuvants [64].

The most advanced ZIKV PIV candidate, ZPIV, uses a Puerto Rican (PR) strain inactivated with 0.05% formalin administered with alum. The ZIKV PIV vaccine induced NAbs and conferred protection against heterologous ZIKV-BR (Brazilian isolated strain) and homologous ZIKV-PR challenges in BALB/c mice [65]. Here, IM administration demonstrated greater efficacy in generating a robust Ab response than subcutaneous injection [65]. However, in rhesus macaques, the opposite pattern was observed, and after challenge, no detectable virus was detected in the analysis panel [66].

The ZPIV vaccine, adjuvanted with Alum, is currently in four phase I clinical trials and are registered as NCT03008122 (two 2.5 mcg doses of ZPIV), NCT02963909 (two 5.0 mcg doses of ZPIV), NCT02952833 (three dose levels 2.5 mcg, 5.0 mcg, and 10 mcg of PIV), and NCT02937233 (three groups divided into different vaccination schedules of 5 mcg of ZPIV administered IM at Week 0 and Week 4; 5 mcg of ZPIV administered IM at Week 0 and Week 2; and a single dose of 5 mcg of ZPIV administered IM at Week 0). In these trials, PIV immunization elicited robust NAbs, however some moderate side effects were noted including headache, fatigue, and malaise [67]. The PIV platform is also being explored for protection against JEV. The PIV vaccine against JEV is also undergoing human clinical trials and is administrated by two or three IM injections and elicits a robust immune response against JEV. Unfortunately, administration of the JEV PIV is fraught with the same moderate adverse effects as ZIKV PIV [68].

### 3.2. DNA Vaccines

DNA-based vaccines are easily designed, manufactured, and are simple to handle and transport [69,70,71]. However, the limiting factor for the use of DNA-based vaccines is their requirement for additional delivery devices such as electroporation to facilitate DNA entry into the cell.

Three different DNA-based ZIKV vaccines have entered clinical trials. VRC-ZKADNA085-00-VP (VRC5288, NCT02840487) is one DNA-based ZIKV vaccine candidate that was constructed using a prME sequence from a French Polynesian isolate (strain H/PF/2013). A second vaccine, named VRC5283, was created from VRC5288 by exchanging the ZIKV prM with an analogous sequence from JEV to improve protein secretion. VRC5283 showed high immunogenicity in mice and NHPs and induced NAb production, which prevented viremia with greater efficiency than VRC5288. During phase I trials, VRC5288 vaccination generated detectable humoral and T-cell mediated responses in 100% of participants [72]. VRC5288 is currently in phase two clinical trials and VRC5283 will soon advance to phase 2 efficacy testing [72].

The third DNA-based candidate, GLS-5700, was developed by GeneOne Life Science/Inovio Pharmaceuticals Inc. and has demonstrated an anti-ZIKV response in animal models and is being investigated as part of two separate phase I clinical trials (NCT02809443 and NCT02887482) [66,73]. GLS-5700 is a consensus DNA vaccine designed to expresses the ZIKV prME in a modified pVAX1 backbone with initial reports showing induction of protective immunity [65,66]. Furthermore, GLS-5700 was also able to prevent ZIKV-induced testes damage and reduced the chronic presence of virus in the male reproductive tract in a mouse model [40]. Further clinical studies will be required to evaluate GLS-5700’s protective efficacy and long-term immunogenicity.

Indeed, DNA vaccines are appropriate for emerging infectious diseases, as this platform is versatile in its ability to target many distinct antigens and said vaccine can be developed and tested quickly [17]. For example, only seven months was required from in silico design of the consensus GLS-5700 antigen to commencement of the phase I clinical trial [73].

### 3.3. RNA Vaccines

The new mRNA vaccines bring fast, precise, and promising alternatives for new therapies to combat several human diseases, pathogens, and cancers. However, delivery of a mRNA vaccine still requires many technological and scientific advancements in order to improve their in vivo delivery [73]. Chahal et al. designed a modified dendrimer nanoparticle (MDNP) with a prME ZIKV RNA replicon. Following vaccination of C57BL/6 mice, induction of anti-ZIKV IgG was noted. Ex vivo vaccination analysis showed that immunization promoted the peptide antigen presentation through MHC-I. This promoted a strong CD8+ T cell response [74]. Pardi et al. developed a lipid nanoparticle (LNP) to encapsulate modified mRNA containing the prME sequences from the H/KPF/2013 ZIKV strain containing a modified nucleoside 1-methylpseudouridine, which increased mRNA translation in vivo [67]. The data showed the generation of protective immunity in mice and NHP models after a single immunization [75]. Richner et al. used the same LPN method to package modified mRNA containing the prME sequence from the Micronesia 2007 ZIKV strain. Administration via IM injection in immunocompromised (Ifnar1^−/−^, Ifngr^−/−^ AG129) and immunocompetent (C57BL/6) mouse strains induced protective immunity with long-term, high-titer NAbs [41]. Furthermore, this vaccine blocked vertical transmission and prevented fetal damage [76]. This vaccine is currently undergoing phase I/II clinical trials (NCT03014089).

### 3.4. Choice of Vaccine Target and Consequences

ZIKV shares several features with DENV (58.1% to 58.9% identity with the DENV2 polyprotein) and the 1952 Nigerian Chuku strain of Spondweni virus, with the open reading frames (ORFs) exhibiting a 61–68% nucleotide and 64–72% amino acid identity [77,78]. Due to the presence of several identical epitopes, many flaviviruses are potentially capable of eliciting cross-reactive, and therefore, cross-protective Abs. The identification of cross-reactive monoclonal Abs (mAbs) from patients exposed to various flaviviruses presents an important progress in the development of novel vaccines [79]. However, cross-reactive mAbs induced by infective virus may result in the induction of poorly neutralizing Abs or result in elevated rates of infection and subsequently exacerbation of disease severity during subsequent reinfection [80,81]. This is due paradoxically to the mAb facilitation of viral entry via Fc receptor-γ:mAb interactions, which promotes viral entry and elevated disease severity [82,83,84]. This phenomenon is termed ADE and occurs when the cross-reactive mAb subneutralizes the virus and facilitates viral uptake by permissive cells. It is therefore essential that optimal neutralization targets and strategies are taken into consideration when developing a vaccine against ZIKV to avoid the complications seen in ADE as ADE may contribute to development of congenital ZIKV disease and enhanced ZIKV infection [79,85]. Furthermore, in vitro experiments suggest that DENV poly- and monoclonal Abs enhance ZIKV infection and ZIKV Abs facilitate Dengue serotype 2 infection [86,87,88]. It is therefore essential that vaccine developers fully understand how ADE may affect a vaccine’s immune response during subsequent viral infections.

Brault et al. described a method to evade ADE complications by expressing the ZIKV NS1 protein in a MVA vector in view of the fact that the major immunogen that promotes cross-reaction is the prME proteins [22].

Richner et al. also developed a novel strategy to mitigate the risk of ADE. Richner et al., generated a modified mRNA that destroyed the conserved fusion-loop epitope found in the E protein and encoded the IgE human signal sequence. The IgE sequence can contribute to translational efficiency and can enhance the stability of mRNA [41,89]. This ZIKV-prME fusion loop mutant was subsequently packaged into lipid nanoparticles and used to immunize mice. Removal of the fusion loop decreased the production of antibodies capable of enhancing DENV infection in cells and mice [41]. These recent advancements in the prevention of ADE further underscore the importance of target antigen choice, specificity, and relative immunogenicity as prime factors in the development of vaccines against ZIKV.

## 4. Other *Flavivirus* Vaccines

The likelihood of a safe, effective, and low-cost vaccine for Zika virus reaching licensure will increase if those developing vaccines learn from the successes and mistakes made in the development of vaccines against other *Flaviviruses*. There are currently a number of licensed *Flaviviruses* vaccines and a number of vaccines in clinical development. The LAV vaccine for Yellow fever, YFV 17D, was the first *Flavivirus* vaccine ever produced and is considered a success in the field with more than 500 million people having received the vaccine and due to its ability to induce protective immunity in ~99% of those vaccinated [90]. There are some draw backs to the YFV 17D LAV vaccine however, including numerous vaccine-associated adverse events, such as neurotropic disease and severe allergic reactions due to the presence of chicken protein in the vaccine from the inactivation process [91]. To avoid these issues, an inactivated cell-culture based version of 17D, XRX-001, is in development. Monath et al. has demonstrated both preclinically and clinically that XRX-001 is relatively safe, induces a suitable immune response, and is effective at protecting animal models against Yellow fever virus infection [92,93].

A purified, formalin-inactivated vaccine against JEV, CC_JEV, is also in late stage clinical development [94]. This vaccine has a promising immunogenicity and safety profile [95] and a similar version of this vaccine has been approved for use in horses. Sanofi Pasteur has also developed a JEV vaccine called ChimeriVax-JE that consists of a live, attenuated Yellow fever virus 17D in which the envelope protein has been replaced with the JEV envelop. This vaccine has demonstrated similar safety and immunogenicity profiles as YFV 17D [43,96].

The relative ease in which vaccines have been developed against YFV and JEV may be in part due to the fact that only a single serotype exists for these viruses, thus lending hope to the idea that a vaccine against the single serotype ZIKV may also be possible. A vaccine against DENV, with four serotypes, has had a much more troublesome road. As mentioned in Section 2.3 of this review, there are DNA-based vaccines against DENV that have demonstrated some immunogenicity and protective efficacy in mice [47,48], although these vaccines have not progressed into the clinic and thus far, no single DNA-based vaccine has been developed to protect against all four serotypes.

The dengue vaccine CYD-TDV (Dengvaxia^®^), expresses the prME sequences from DENV-1, DENV-2, DENV-3, and DENV-4 in an YFV 17D backbone, and is thus tetravalent. Based on initial phase III clinical trial data that demonstrated favorable safety and immunogenicity results [97], this Sanofi Pasteur product was licensed in 20 countries. Unfortunately, recent follow-up studies by the company have demonstrated that the vaccine performs differently in seronegative versus seropositive individuals in that there is an increased risk of hospitalization and severe dengue in seronegative individuals starting about 30 months after vaccination [98]. As such, the World Health Organization has changed their recommendations for this vaccine [99].

Despite the draw backs experienced in the DENV field, there is no data to date that would suggest that similar problems will be encountered in the development of a ZIKV vaccine.

## 5. Conclusions

The *Flavivirus* genus is compose of more than 70 viruses, most of which are transmitted by mosquitoes and are present in distinct geographical locations. These viruses use different natural hosts and specific vectors making them difficult to control and making vaccination a cost-effective intervention to minimize the impact of future outbreaks.

Several efforts to find a safe vaccine to prevent ZIKV infection and induce protective immunity with a single administration have been made by scientists around the world. Most vaccine platforms in development aim to find a method to prevent vertical transmission from mother to fetus, which could otherwise result in congenital ZIKV syndrome. Even with the development of vaccines focused on prevention of vertical transmission and fetal abnormalities, further efforts should be made to elucidate the mechanism(s) by which ZIKV elicits an auto-reactive state manifested as syndromes such as Guillain–Barré.

Nevertheless, the rapid development of a safe, tolerable, and effective vaccine is imperative due to the potential for future outbreaks. Numerous platforms have been tested both preclinically and clinically and have generated encouraging results. The LAV and PIV platforms have demonstrated a robust induction of protective immunity, however, the safety profile of these platforms is not ideal for the vaccination of pregnant women. The DNA and RNA platforms under development are a safer option for this target demographic and have demonstrated consistent immune responses. Additional advantages to the DNA- and RNA-based vaccine platforms is their target antigen versatility and the speed in which they can be developed. Should there be a future ZIKV outbreak with a strain not currently covered by existing vaccines, these platforms have the greatest potential for quick adaptation and clinical evaluation.

In addition, the choice of the vaccine immunogen plays a significant role in the robustness of the immune response. The ZIKV structural proteins C, prM, and E, and nonstructural proteins NS1 and NS5 were selected as candidate targets within several vaccines platforms. Several demonstrated protection against various ZIKV strains. Furthermore, some immunogens were selected and designed to avoid the development of ADE, which is an important complication related to flavivirus infection.

In conclusion, an ideal ZIKV vaccine will consist of a platform and immunogen capable of conferring long-lasting protection against all ZIKV strains to the greatest number of individuals in a cost-effective manner. The continued development and testing of the promising vaccine candidates outlined in this review will most likely result in at least one vaccine reaching licensure.
